# Perhexiline promotes HER3 ablation through receptor internalization and inhibits tumor growth

**DOI:** 10.1186/s13058-015-0528-9

**Published:** 2015-02-15

**Authors:** Xiu-Rong Ren, Jiangbo Wang, Takuya Osada, Robert A Mook, Michael A Morse, Larry S Barak, Herbert Kim Lyerly, Wei Chen

**Affiliations:** Department of Medicine, Duke University Medical Center, 595 Lasalle street, Durham, NC 27710 USA; Duke Comprehensive Cancer Center, Department of Surgery, Duke University Medical Center, 200 Trent Drive, Durham, NC 27710 USA; Department of Cell Biology, Duke University Medical Center, 483 Clin & Res Labs, Durham, NC 27710 USA

## Abstract

**Introduction:**

Human epidermal growth factor receptor HER3 has been implicated in promoting the aggressiveness and metastatic potential of breast cancer. Upregulation of HER3 has been found to be a major mechanism underlying drug resistance to EGFR and HER2 tyrosine kinase inhibitors and to endocrine therapy in the treatment of breast cancer. Thus, agents that reduce HER3 expression at the plasma membrane may synergize with current therapies and offer a novel therapeutic strategy to improve treatment.

**Methods:**

We devised an image-based screening platform using membrane localized HER3-YFP to identify small molecules that promote HER3 internalization and degradation. *In vitro* and *in vivo* tumor models were used to characterize the signaling effects of perhexiline, an anti-anginal drug, identified by the screening platform.

**Results:**

We found perhexiline, an anti-anginal drug, selectively internalized HER3, decreased HER3 expression, and subsequently inhibited signaling downstream of HER3. Consistent with these results, perhexiline inhibited breast cancer cell proliferation *in vitro* and tumor growth *in vivo.*

**Conclusions:**

This is the first demonstration that HER3 can be targeted with small molecules by eliminating it from the cell membrane. The novel approach used here led to the discovery that perhexiline ablates HER3 expression, and offers an opportunity to identify HER3 ablation modulators as innovative therapeutics to improve survival in breast cancer patients.

**Electronic supplementary material:**

The online version of this article (doi:10.1186/s13058-015-0528-9) contains supplementary material, which is available to authorized users.

## Introduction

Human epidermal growth factor receptor 3 (HER3) is a single membrane-spanning receptor that exerts its function through heterodimerization with other HER family receptors [1-3]. Activation of HER3-containing heterodimers leads to HER3 phosphorylation and subsequent activation of signaling pathways, including phosphatidylinositol 3-kinase (PI3K)/Akt and RAF/MEK/extracellular signal-regulated protein kinase (ERK) pathways, that drive tumor cell proliferation and promote survival [2,4]. Increasing evidence has identified HER3 as one of the most potent oncogenic factors in promoting breast cancer tumorigenesis [5]. In animal models of breast cancer driven by HER2, HER3 expression and phosphorylation are upregulated [6,7]. Knockout of HER3 impairs the ability of HER2 to induce tumor formation in mouse mammary tumor virus-HER2 (MMTV-HER2)-driven mouse models [8]. In addition, overexpression of HER3 has been shown to significantly enhance the invasiveness of breast cancer cells. Specifically, HER3 promotes invasion and metastasis through its ability to activate the PI3K pathway [9]. The clinical impact of HER3 is indicated by the observation that increased HER3 expression and the detection HER2/HER3 dimers have prognostic significance in breast cancer [10,11].

Therapies targeting epidermal growth factor receptor (EGFR) and HER2 have been extensively developed. Unfortunately, clinical efficacy is not satisfactory as drug resistance reduces durable responses. Upregulation of HER3 has been found as one of the major mechanisms underlying drug resistance to EGFR and HER2 tyrosine kinase inhibitors (for example lapatinib, gefitinib, erlotinib) and to endocrine therapy in the treatment of breast cancer [12-14]. For example, it has been shown that the expression of HER3 ligand heregulin (HRG) as well as activation of HER3 signaling is involved in resistance to anti-estrogen therapies *in vitro* and *in vivo* [15-17]. It has been suggested that effective therapies against HER2 require simultaneous targeting of HER3 [18]. Thus, mounting evidence highlights the importance of targeting HER3 to decrease breast cancer mortality [3].

In this report, we engineered an image-based screening platform using membrane localized HER3-yellow fluorescent protein (YFP) to identify small molecules that promote HER3 internalization and degradation. Using this platform, we screened a library of Food and Drug Administration (FDA) and foreign regulatory agency-approved drugs, and identified that perhexiline, an anti-anginal drug that inhibits mitochondrial carnitine palmitoyltransferase I (CPT-1) [19], promotes HER3 internalization and downregulation, inhibits signaling downstream of HER3, and inhibits cancer cell proliferation *in vitro* and *in vivo*.

## Materials and methods

### Reagents and antibodies

Perhexiline maleate salt was purchased from Sigma-Aldrich (St. Louis, MO, USA). Neuregulin was purchased from R&D Systems (Minneapolis, MN, USA). The following antibodies were purchased from Santa Cruz Biotechnology (Dallas, TX, USA): HER2 (3B5), HER3 (C-17), and ubiquitin. The pAkt (Ser473), pERK1/2, total EGFR, and Alexa Fluor™ 488 Conjugate Flag antibodies were purchased from Cell Signaling (Beverly, MA, USA). The antibodies were used at a 1:500 dilution in Western blotting. LysoTracker™ Red DND-99 was purchased from Invitrogen (Grand Island, NY, USA).

### Cell culture

HEK293 and U2OS cells were cultured in Minimum Essential Medium (MEM), SK-BR-3 cells were cultured in McCoy’s 5A medium, and MDA-MB-468 cells were cultured in Leibovitz’s L-15 medium. AU565 and BT474 cells were cultured in RPMI-1640 medium. All media were supplemented with 10% fetal bovine serum (FBS, Atlanta Biologicals, Lawrenceville, GA, USA), 200 U/ml penicillin, and 50 ng/ml streptomycin (Invitrogen, Grand Island, NY, USA). Cells were grown at 37°C in 5% CO_2_ except for the MDA-MB-468 cells that were grown at 37°C without CO_2_. All cell lines were purchased from American Type Culture Collection (Manassas, VA, USA).

### Expression constructs

The cDNA clone of human HER3 (pCMV-sport6-ERBB3, IMAGE: 6147464) was obtained from the Mammalian Gene Collection (MGC) through Open Biosystems (GE Dharmacon, Lafayette, CO, USA). To generate the C-terminal YFP-tagged HER3 (HER3-YFP), the coding region of HER3 was amplified using polymerase chain reaction (PCR) and subcloned in-frame into pcDNA3.1-mYFP vector (a gift from Roger Y Tsien, University of California at San Diego, USA). The primers used for PCR amplification were 5′-GGGGTACCGGAGTCATGAGGGCGAACGACGCTC −3′ and 5′-ATAAGAATGCGGCCGCGTTCTCTGGGCATTAGCCTTGGG −3′, and the HER3 fragment was cloned into the *Kpn I* and *Not I* sites on the vector. In order to delete the nuclear localization sequence (NLS2, ‘RRRR’) in HER3, site-directed mutagenesis experiments were performed using HER3-YFP as the template, and the primers used were: 5′-GAGTATGAATACATGAACCACAGTCCACCTCATCCC −3′ and 5′-GGGATGAGGTGGACTGTGGTTCATGTATTCATACTC −3′. To generate the Flag-tagged HER3ΔNLS2 construct, the coding sequence was amplified by PCR (primers used were: 5′-GGGGTACCGAGGGCGAACGACGCTCTG-3′and 5′-GCTCTAGATTACGTTCTCTGGGCATTAGC-3′) and subcloned into the *Kpn I* and *Xba I* sites on the pFlag-CMV3 vector (Sigma-Aldrich, St Louis, MO, USA). All constructs were verified by sequencing.

### Imaging-based primary screening assay

Primary screening assays were performed as previously described [20,21]. Briefly, U2OS cells stably expressing HER3ΔNLS2-YFP were treated with compounds from a library containing approximately 1,200 FDA and foreign regulatory agency-approved drugs and drug-like tool compounds (Prestwick Chemical Illkirch-Graffenstaden, France). Cells were incubated with each compound for 6 hours at 37°C prior to fixation in phosphate-buffered saline (PBS) containing 4% paraformaldehyde and 0.002% of the fluorescent nuclear stain DRAQ5. Plates were stored at 4°C until analysis on an ImageXpress Ultra high-throughput imaging system (Molecular Devices, Sunnyvale, CA, USA) equipped with a 488 nm argon laser for imaging GFP and a 568 nm krypton laser for imaging DRAQ5. All imaging data were verified by visual inspection and a Z′ factor of 0.44 was calculated for the robustness of the assay.

### Immunofluorescence staining and imaging analysis

U2OS cells stably expressing HER3ΔNLS2-YFP plated on 35-mm, poly-D-lysine-coated, glass-bottom microwell dishes (MatTek Cultureware, Ashland, MA, USA) were treated with dimethyl sulfoxide (DMSO) or perhexiline for the indicated time at 37°C and followed by fixation with 4% paraformaldehyde. HEK293 cells grown in microwell dishes were transfected (Fugene6; Roche Diagnostics Corp., Indianapolis, IN, USA) with Flag-HER3ΔNLS2, and 24 hours post-transfection cells were incubated with Alexa Fluor™ 488 Conjugate Flag antibody in culture medium on ice for 30 minutes. After washing out unbound antibodies, cells were incubated with perhexiline or DMSO in culture medium at 37°C for 1 hour followed by fixation. To detect endogenous HER3 receptors, MDA-MB-468 cells were allowed to grow for 24 hours and then treated with DMSO or perhexiline for the indicated time at 37°C before fixation in 4% paraformaldehyde. Fixed cells were permeabilized and blocked in blocking buffer (5% bovine serum albumin (BSA) with 0.2% saponin in PBS) for 20 minutes at room temperature and washed in PBS. Where indicated, cells were incubated with HER3 antibody in blocking buffer for 1 hour at room temperature and subsequently incubated with the Alexa Fluor™ 488-conjugated goat anti-rabbit secondary antibody (Invitrogen, Grand Island, NY, USA) in blocking buffer for 1 hour at room temperature. The slides were mounted in mounting medium (Vector Laboratories, Inc., Burlingame, CA, USA) and examined using a LSM 510-Meta confocal microscope (Carl Zeiss, Thornwood, NY, USA) equipped with 40× and 100× apo chromat objectives. YFP was excited using a 488-nm argon laser line. Images were processed using the LSM software Image Browser (Carl Zeiss, Thornwood, NY, USA).

### Assay of HER3 degradation and ubiquitination

MDA-MB-468 or SK-BR-3 cells seeded into 6-well plates (1.5 × 10^5^ cells/well) were allowed to grow for 24 hours in the complete growth medium. Cells were subsequently treated with DMSO or perhexiline (10 μM) for the indicated time. Cell lysates were prepared in 2 × SDS sample buffer and subjected to Western blotting analysis. For ubiquitination assays, cells cultured and treated as described were collected into glycerol lysis buffer (50 mM Hepes, 250 mM NaCl, 0.5% NP40, 10% glycerol, and 5 mM ethylenediamine tetraacetic acid). Cell lysates were incubated with agarose-conjugated anti-HER3 antibody overnight, washed three times with the glycerol lysis buffer, and subjected to Western blotting analysis.

### Western blotting

The protein samples were subjected to SDS-PAGE using 4 to 12% Novex™ Tris-Glycine Gels (Invitrogen, Grand Island, NY, USA), transferred to nitrocellulose membranes (Bio-Rad Laboratories, Hercules, CA, USA) blocked with 5% nonfat milk powder in TBS-0.2% Tween-20 for 30 minutes, followed by incubation with primary antibodies and then horseradish peroxidase-conjugated secondary antibodies (Amersham Biosciences, Piscataway, NJ, USA). The ECL signals were detected using SuperSignal substrate (Pierce Biotechnology, Rockford, IL, USA) and quantified using ChemiDoc™ MP Imaging System (Bio-Rad Laboratories, Hercules, CA, USA).

### Cell proliferation assay

The breast cancer cell lines MDA-MB-468, SK-BR-3, AU565, and BT474 were used in the cell proliferation assay. The cells were plated at 3,000 cells per well into 96-well plates and treated with compounds for 72 hours, at which point the cell proliferation was measured using the colorimetric MTS assay (Promega, Madison, WI, USA). For the dose–response assays, the cells were treated with perhexiline ranging from 0.5 to 10 μM.

To examine the combinational effect of perhexiline and lapatinib, the cells were treated with perhexiline and lapatinib alone or in combination. For the MDA-MB-468 cells, the ratio of perhexiline to lapatinib was 1:1. For the SK-BR-3, AU565, and BT474 cells, the ratio was 20:1. Experiments were performed in three replicates and performed three times. Values were normalized as a percentage of DMSO-treated cells. The Combination Index (CI), which quantifies the degree of synergism in a drug combination, was obtained using the method of Chou and Martin in the software CompuSyn.

### Testing the antitumor effect of perhexiline *in vivo*

All animal experiments were followed animal protocol A294-11-11, which was reviewed and approved by the Duke Institutional Animal Care and Use Committee. MDA-MB-468 tumor cells (1 × 10^6^/mouse) were injected into the flank of SCID mice on day 0. On day 7, oral gavage treatment with perhexiline (0, 400 mg/kg) was initiated, and repeated for 5 days a week for 4 weeks. Tumor diameter was measured every 3 to 4 days. Each group consisted of five mice. Student’s *t* test was used to analyze differences in tumor volumes at each perhexiline concentration compared to vehicle control. Differences at *P* <0.05 were considered statistically significant.

## Results

### Perhexiline promotes HER3 internalization and degradation

**Figure 1:**
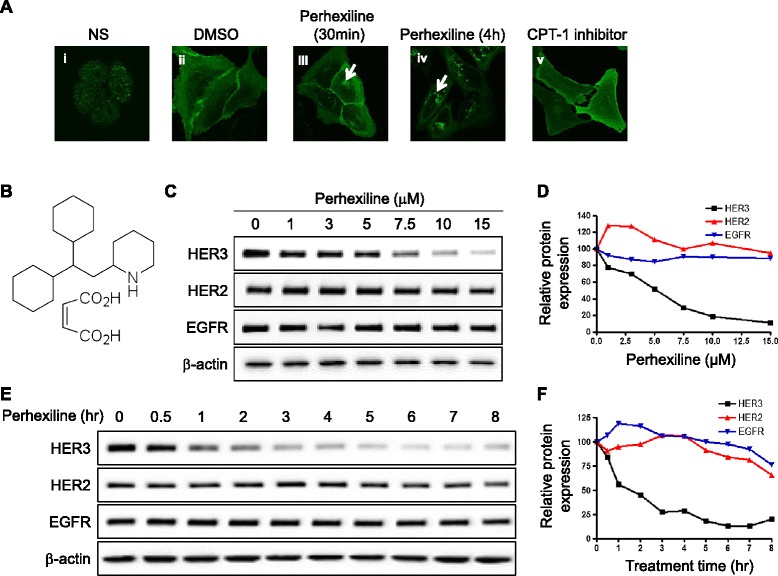
**Identification of perhexiline as a novel agent that promotes HER3 internalization and degradation. (A)** Perhexiline promotes HER3ΔNLS2-YFP internalization. As a control to HER3ΔNLS2-YFP cellular distribution, endogenous HER3 expression in MDA-MB-468 cells was detected by immunofluorescence staining (i). U2OS cells stably expressing HER3ΔNLS2-YFP were used to screen for compounds that promote HER3 internalization. Representative confocal images shown in panels (ii to v) were taken from cells expressing HER3ΔNLS2-YFP that were treated with DMSO, 10 μM perhexiline for 30 minutes and 4 hours, and 20 μM CPT-1 inhibitor etomoxir for 4 hours, respectively. Arrows indicate intracellularly localized puncta of internalized HER3ΔNLS2-YFP receptors. **(B)** Structure of perhexiline maleate. **(C)** Perhexiline induces dose-dependent downregulation of endogenous HER3 receptors. Cell lysates prepared from SK-BR-3 cells treated with different concentrations of perhexiline for 8 hours were analyzed for the expression of HER3, HER2, and EGFR. β-actin was used as a loading control. **(D)** Quantification of HER3, HER2, EGFR protein expression following perhexiline treatment. Western blots shown in **(C)** were quantified by normalizing to β-actin. **(E)** Time course of perhexiline-induced downregulation of endogenous HER3 in SK-BR-3 cells. Cells treated with 10 μM perhexiline for the indicated time were analyzed for endogenous HER3, HER2, and EGFR expression. **(F)** Quantification of HER3, HER2, EGFR protein expression following perhexiline treatment. Western blots shown in **(E)** were quantified by normalizing to β-actin. CPT-1, mitochondrial carnitine palmitoyltransferase-1; DMSO, dimethyl sulfoxide; EGFR, epidermal growth factor receptor; HER3, human epidermal growth factor receptor 3.

In contrast to the other members of the HER family of receptor kinases, the HER3 receptor is not an active kinase [1]. As a result, strategies targeting the kinase activity to develop small molecule therapeutics against HER3 similar to those employed to develop small molecules against EGFR and HER2, are not applicable. To overcome this hurdle, we developed an imaging-based high-throughput screening strategy to search for small molecules that promote HER3 internalization based on the notion that receptor internalization often results in loss of receptor function. Screening compound libraries for their effects on membrane trafficking has proven to be a highly rewarding strategy for identifying inhibitors and activators of other enigmatic membrane receptors such as Frizzled and Smoothened [20,21]. Since endogenous HER3 receptors were expressed at the plasma membrane, the cytosol, and the nucleus in MDA-MB-468 cells (Figure 1A, panel i), therefore in this report, we utilized an YFP-tagged HER3ΔNLS2 (HER3ΔNLS2-YFP) expression construct, with the C-terminal nuclear localization sequence (NLS2, a four Arg-repeat) [22] removed in order to enhance membrane localization. We found that this mutant receptor functions similarly as wild-type (WT) HER3 in promoting downstream signaling upon stimulation with neuregulin β1 (NRGβ1) (Figure S1 in Additional file 1). Stable U2OS cells were generated to express HER3ΔNLS2-YFP and the receptors were predominantly expressed at the cell surface basally (Figure 1A, panel ii), which is different from endogenous HER3 expression throughout the cell (Figure 1A, panel i). Using this cell line, we screened the Prestwick Chemicals Library containing FDA and foreign regulatory agency-approved drugs, and discovered perhexiline maleate (Figure 1B) induced HER3 receptor internalization (Figure 1A, panels iii and iv). Perhexiline is a drug once used to treat angina (Figure 1B) [19]. The mechanism responsible for its anti-angina activity is believed to result from inhibition of mitochondrial CPT-1. To test whether inhibition of CPT-1 induced HER3 receptor internalization, we tested the effect of another CPT-1 inhibitor, etomoxir, on HER3 internalization. Our result showed that etomoxir had no effect on HER3 localization (Figure 1A, panel v) suggesting perhexiline operates through a mechanism independent of its ability to inhibit CPT-1 [23].

Since receptor internalization often leads to degradation of receptor proteins, we next examined the effect of perhexiline on the level of endogenous HER3 receptors. As shown in Figure 1C and D, treating SK-BR-3 cells with increasing concentrations of perhexiline for 8 hours resulted in a dose-dependent downregulation of HER3 protein. Interestingly, this effect is specific for HER3 only as the expression of receptors in the same family, including EGFR and HER2, were not affected by perhexiline treatment (Figure 1C and D). Moreover, perhexiline (10 μM)-mediated downregulation of HER3 was observed after 30 minutes and less than 10% of HER3 remained at the end of the 8-hour treatment (Figure 1E and F). A similar effect of perhexiline on decreasing HER3 expression was detected in triple-negative breast cancer MDA-MB-468 cells that express EGFR and HER3, but not HER2 (Figure S2 A-D in Additional file 2). We also observed general cell toxicity when cells were treated at 15 μM perhexiline. Perhexiline also promotes the degradation of phosphorylated HER3, an active form for HER3 signaling (Figure S2E in Additional file 2) [9].

### Perhexiline internalizes cell surface-expressed HER3 and induces receptor ubiquitination

**Figure 2:**
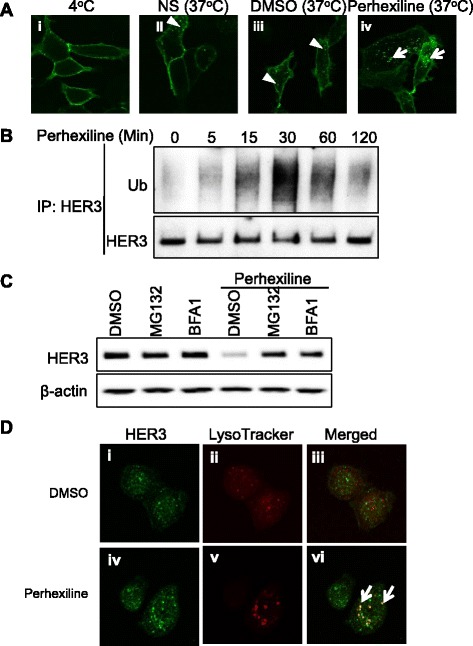
**Perhexiline promotes cell surface-expressed Flag-HER3ΔNLS2 receptor internalization and the ubiquitination-mediated degradation of HER3 receptors through lysosome and proteasome. (A)** Cell surface-expressed Flag-HER3ΔNLS2 receptors are internalized upon perhexiline treatment. HEK293 cells transfected with Flag-HER3ΔNLS2 were incubated with the anti-FLAG antibody on ice for 30 minutes (i) and subsequently incubated at 37°C for 1 hour untreated (ii) or in the presence of DMSO or perhexiline (10 μM) (iii, iv). Cells were fixed and localization patterns of Flag-HER3ΔNLS2 were visualized using confocal microscopy. Internalized Flag-HER3ΔNLS2 vesicles were shown as indicated by arrowheads in DMSO-treated cells whereas arrows indicate large puncta of internalized receptors in perhexiline-treated cells. **(B)** Perhexiline induces HER3 ubiquitination. MDA-MB-468 cells were treated with perhexiline (10 μM) for the indicated time. The endogenous HER3 was immunoprecipitated and the amount of ubiquitination and total HER3 were detected using the anti-ubiquitin and HER3 antibodies, respectively. **(C)** Perhexiline-induced downregulation of HER3 is blocked by proteasome and lysosome inhibitors. MDA-MB-468 cells were treated with DMSO, MG132 (10 μM), or BFA1 (30 nM) in the absence or presence of perhexiline (10 μM) for 8 hours. The endogenous HER3 was detected by the anti-HER3 antibody. **(D)** Perhexiline induces the redistribution of endogenous HER3 to the lysosome. MDA-MB-468 cells treated with DMSO (i to iii) or 10 μM perhexiline (iv to vi) for 2 hours were fixed and analyzed using confocal microscopy. The localization of HER3 and the lysosome was visualized using the anti-HER3 antibody (green) and LysoTracker (red), respectively. Co-localization of HER3 (green) with lysosome (visualized by LysoTracker Red) was observed in perhexiline-treated cells as yellow puncta marked by arrows. DMSO, dimethyl sulfoxide; EGFR, epidermal growth factor receptor; HER3, human epidermal growth factor receptor 3.

Our data showed that perhexiline stimulates HER3 internalization and degradation. To further prove that the internalized receptor was from the cell surface, we generated an N-terminal Flag-tagged HER3ΔNLS2 construct. Live cells expressing surface-localized Flag-HER3ΔNLS2 receptors were labeled on ice with fluorescence-conjugated Flag antibody to prevent internalization of the receptor (Figure 2A, panel i). Raising the temperature to 37°C caused a small amount of Flag-HER3ΔNLS2 receptors to internalize as indicated by the redistribution of labeled receptors to the cytoplasm in untreated or DMSO-treated cells (Figure 2A, panels ii and iii). However, more internalized receptors were observed in cells treated with perhexiline for 1 hour, and these internalized receptors formed larger puncta inside the cells (Figure 2A, panel iv).

We next determined if perhexiline induces HER3 degradation by promoting ubiquitination of the receptor. To this end, MDA-MB-468 cells were treated with perhexiline over the time course of 2 hours, and endogenous HER3 receptors were immunoprecipitated from the cells. We found that perhexiline treatment was accompanied by a rapid increase in the level of HER3 ubiquitination (Figure 2B). Furthermore, perhexiline-induced degradation of HER3 was partially rescued by pretreating cells with MG132, a proteasome inhibitor, or bafilomycin A1 (BFA1), a lysosome inhibitor (Figure 2C), suggesting that perhexiline promotes both proteasome- and lysosome-dependent degradation of ubiquitinated HER3 receptors. Furthermore, despite the fact that endogenous HER3 receptors were expressed at the plasma membrane, the cytosol, and the nucleus in MDA-MB-468 cells, treating cells with perhexiline resulted in the redistribution of endogenous HER3 to the lysosomal compartment. The internalized receptors were co-localized with lysosomes in perhexiline-treated cells (Figure 2D). Similarly, U2OS cells expressing HER3-ΔNLS2-YFP were treated with DMSO (i to iii) or perhexiline (iv to vi) and the internalized HER3-ΔNLS2-YFP receptors were co-localized with lysosomes, confirming that perhexiline induces HER3 degradation via the lysosome pathway (Figure S3 in Additional file 3).

### Perhexiline inhibits neuregulin-mediated signaling activation and breast cancer cell proliferation

**Figure 3:**
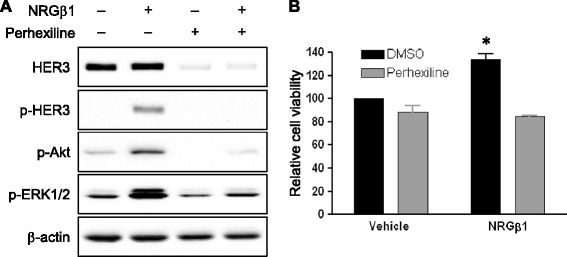
**Perhexiline treatment inhibits neuregulin-mediated activation of downstream signaling and cell proliferation. (A)** MDA-MB-468 cells were cultured in the absence or presence of perhexiline (10 μM) for 6 hours, and subsequently stimulated with DMSO or NRGβ1 (10 ng/ml) for 10 minutes. Cell lysates were analyzed for the phosphorylation of HER3 (Tyr1289), Akt and ERK1/2 as well as total HER3 expression. **(B)** Perhexiline inhibits NRGβ1-induced cell growth. MB-MDA-468 cells were treated for 72 hours with vehicle (0.2% BSA in PBS) or NRGβ1 (10 ng/ml) in the presence of DMSO or perhexiline (2 μm). Cell growth was measured using the MTS assay. NRGβ1 treatment resulted in 33.5% increase of cell proliferation. Perhexiline inhibits 11.5% cells in vehicle media and 36.3% cell growth in NRGβ1 media. Data are presented as the mean ± SEM (n = 3). The statistical significance between DMSO control and perhexiline treatment in vehicle media or NRGβ1 media was analyzed by a two-tailed Student’s *t* test, (with ^*^ *P* <0.05 defined as significant compared to the control group with only NRGβ1 treatment). BSA, bovine serum albumin; DMSO, dimethyl sulfoxide; ERK, extracellular signal-regulated protein kinase; HER3, human epidermal growth factor receptor 3; NRGβ1, neuregulin β1; PBS, phosphate-buffered saline; SEM, standard error of the mean.

To determine the effect of perhexiline on HER3-mediated oncogenic signaling in breast cancer cells, serum-starved MDA-MB-468 cells were pretreated with perhexiline and subsequently stimulated with NRGβ1. As shown in Figure 3A, perhexiline treatment significantly decreased NRGβ1-induced phosphorylation of Akt and ERK. This inhibition of signaling coincided with perhexiline-mediated downregulation of HER3 (Figure 3A). In addition, MTS assays were performed to assess the effect of perhexiline on cell proliferation. Results showed that NRGβ1 treatment resulted in a 33.5% increase in cell proliferation consistent with HER3’s role in promoting tumor growth. Treating cells with perhexiline decreased cell proliferation by 11.5% and 36.3% in serum-free and NRGβ1-containing medium, respectively (Figure 3B).

**Figure 4:**
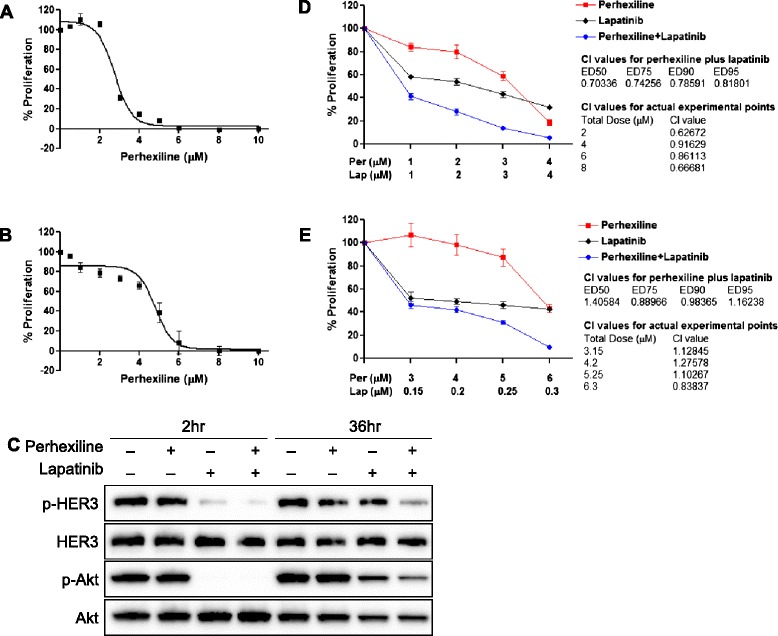
**Perhexiline inhibits breast cancer cell proliferation and functions synergistically with lapatinib. (A-B)** MDA-MB-468 **(A)** and SK-BR-3 **(B)** cells were treated with different concentrations of perhexiline for 72 hours, and cell viability was measured using the MTS assay. Each experimental point was performed in triplicate, and results represent mean ± SEM of three independent experiments. **(C)** The synergistic effect of perhexiline and lapatinib on inhibiting HER3-mediated Akt signaling. MDA-MB 468 cells treated with 5 μM perhexiline alone or in combination with 100 nM lapatinib for 2 or 36 hours. Cell lysates were analyzed for the phosphorylation status of endogenous HER3 and Akt as well as total HER3 and Akt expression. **(D-E)** Perhexiline and lapatinib synergistically inhibit MDA-MB-468 **(D)** and SK-BR-3 **(E)** cell growth. Cells were treated with increasing concentrations of perhexiline and lapatinib alone or in combination for 72 hours. MTS assays were performed to measure cell viability. Values are expressed as a percentage of DMSO-treated cells. Results are presented as the mean ± SEM. Combinational index (CI) quantifies the degree of synergism in a drug combination. CI is obtained using the method of Chou and Martin in the software CompuSyn. CI <1 indicates drug synergy. ED50, ED75, ED90 and ED95 are the effective doses at which 50%, 75%, 90% and 95% cells are killed, respectively. DMSO, dimethyl sulfoxide; HER3, human epidermal growth factor receptor 3; SEM, standard error of the mean.

To further determine the effect of perhexiline on breast cancer cell proliferation, MDA-MB-468 and SK-BR-3 cells were treated with different concentrations of perhexiline for 72 hours in regular growth medium. Perhexiline treatment effectively inhibited cell proliferation in both cell lines with IC_50_ of 2.7 ± 0.07 and 4.8 ± 0.2 μM for MDA-MB-468 and SK-BR-3, respectively (Figure 4A-B). These IC_50_ values were similar to those needed to induce internalization and degradation of HER3 receptors. Given upregulation of HER3 has been found to be a major mechanism underlying drug resistance to EGFR and HER2 tyrosine kinase inhibitors (for example lapatinib, gefitinib, erlotinib) [12-14], we next assessed the effect of perhexiline combined with lapatinib, an agent commonly used to treat HER2-positive breast cancer. Treatment of MDA-MB-468 cells with 100 nM lapatinib for 2 hours led to decreased phosphorylation and activation of HER3 and downstream Akt signaling (Figure 4C). However, lapatinib treatment after 36 hours led to the recovery of HER3 and AKT phosphorylation, a result similar to the original report describing lapatinib resistance mediated by HER3 (Figure 4C) [12]. In contrast, the inhibition of HER3 and downstream Akt signaling remained effective in cells treated with a combination of 5 μM perhexiline and 100 nM lapatinib, suggesting that perhexiline can potentially be used to prevent the resistance to HER family tyrosine kinase inhibitors (Figure 4C).

The effect of the combination treatment on cell proliferation was then studied in MDA-MB-468 and SK-BR-3 cells. Combination of perhexiline and lapatinib at each of the four indicated doses (1 to 4 μM) resulted in a more pronounced inhibition in cell proliferation compared to cells treated with perhexiline or lapatinib alone in MDA-MB-468 cells (Figure 4D). Statistical analysis of the combination dose effect [24] demonstrated that combinational index (CI) values were less than 1, indicating perhexiline synergistically enhances the antitumor effect of lapatinib.

The combinational effects of perhexiline and lapatinib on SK-BR-3 cells also resulted in similar inhibition in cell proliferation. The CI values of the 75% and 90% effective doses (ED75, ED90) were less than 1. The CI value for one experimental point was below 1 (Figure 4E).

We chose two additional breast cancer cell lines, AU565 and BT474, to test the inhibitory effect of perhexiline on cell proliferation, both alone and in combination with lapatinib. Perhexiline inhibited cell proliferation in both cell lines with IC_50_ of 6.1 ± 0.07 and 4.0 ± 0.04 μM, respectively (Figure S4A, C in Additional file 4). Perhexiline also synergistically enhanced the antiproliferative effect of lapatinib. For example, in AU565 cells, the CI values at the ED75, ED90 and ED95 as well as at all four experimental combinations were less than 1 (Figure S4B in Additional file 4), suggesting robust synergistic effect. In BT474 cells, however, the CI values were less than 1 at the ED75 and ED90 doses and in only two out of four experimental combinations (Figure S4D in Additional file 4), suggesting the synergy of the combination in BT474 cell line was less robust. These data indicate that perhexiline inhibits breast cancer cells growth and its inhibitory effect is synergized with lapatinib.

### Perhexiline inhibits tumor growth *in vivo*

**Figure 5:**
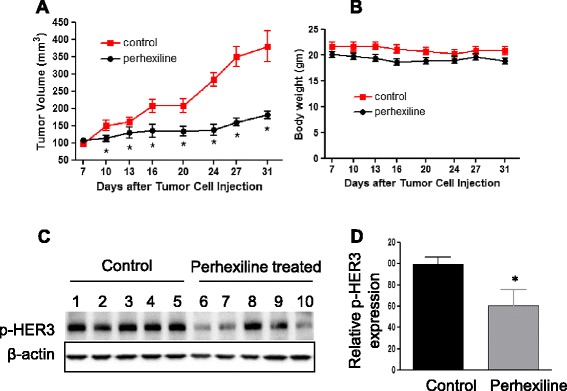
**Perhexiline inhibits the tumorigenesis of MDA-MB-468 cells in NOD/SCID mice. (A)** MDA-MB-468 cells (1 × 10^6^/mouse) were injected to the flank of SCID mice on day 0. On day 7, oral gavage treatment with perhexiline (0, 400 mg/kg) was initiated, and repeated for 5 days a week for 4 weeks. Tumor diameter was measured every 3 to 4 days. Each group consisted of five mice. Date represent mean ± SEM (n = 5, ^*^ *P* <0.05 by a two-tailed Student’s *t* test). **(B)** The body weight was measured at the same time of tumor measurement. Each group consisted of five mice. Data represent mean ± SEM (n = 5, ^*^ *P* <0.05 by a two-tailed Student’s *t* test). **(C)** Tumor samples were collected from all five mice in the control and perhexiline-treated group and processed for Western blotting analysis. The level of phospho-HER3 was detected using the phospho-Tyr1289 antibody. β-actin was used as a loading control. **(D)** Quantitative representation of p-HER3 levels. Western blots shown in **(C)** were quantified by normalizing to β-actin. Data represent mean ± SEM (n = 5, ^*^ *P* <0.05 by a two-tailed Student’s *t* test compared to the control group). HER3, human epidermal growth factor receptor 3; SEM, standard error of the mean.

The antitumor effect of perhexiline was then studied *in vivo*. Triple-negative MDA-MB-468 cells were inoculated subcutaneously into SCID mice and 7 days later, oral gavage perhexiline was initiated and dosed five times a week. As shown in Figure 5A, perhexiline significantly inhibited the growth of MDA-MB-468 tumors. During the course of the treatment with perhexiline, no obvious side effects such as body weight loss were observed in the treated mice (Figure 5B). In addition, tumor masses were excised, homogenized, and immune-blotted for the expression of phosphorylated HER3. Perhexiline-treated tumors showed significantly decreased levels of phosphorylated HER3 compared to control tumors (Figure 5C-D). Taken together, these results indicated that perhexiline inhibits tumor growth and HER3 signaling *in vivo*.

## Discussion

Collectively, we report the development of a novel approach to inhibit HER3 signaling via the application of an imaging-based HER3 internalization assay to identify small molecule inhibitors of HER3-mediated signaling. This robust assay allows high-throughput screening to discover both small molecules and antibodies targeting HER3. Moreover, it overcomes the barrier of developing HER3-targeted small molecule therapies imposed by the fact that HER3 lacks active kinase activity. To our knowledge, perhexiline is the first small molecule identified that promotes selective HER3 degradation and inhibition of HER3-mediated signaling by inducing receptor internalization. Consistent with this mechanism, perhexiline also inhibits breast cancer cell proliferation *in vitro* and tumor growth *in vivo* and synergizes with lapatinib to inhibit breast cancer cell growth.

Increasing evidence has identified HER3 as one of the most potent oncogenic factors in promoting breast cancer tumorigenesis [5]. In animal models of breast cancer driven by HER2, HER3 expression and phosphorylation are upregulated [6,7]. In addition, overexpression of HER3 has been shown to significantly enhance the invasiveness of breast cancer cells. Specifically, HER3 promotes invasion and metastasis through its ability to activate the PI3K pathway [9]. The clinical impact of HER3 is indicated by the observation that increased HER3 expression [10] and the detection HER2/HER3 dimers [11] have prognostic significance in breast cancer.

With the exception of HER3, the HER family of membrane receptors all have intrinsic tyrosine kinase activity. This kinase activity enabled successful small molecule drug discovery approaches by targeting the kinase enzyme activity with inhibitors that bind in the ATP active site. Given HER3 is not an active kinase enzyme, small molecule approaches to inhibit its signaling activity was not considered feasible. Pertuzumab, a monoclonal antibody that blocks dimerization of HER2 and HER3 receptors, was approved by the FDA for concurrent use in combination with trastuzumab and docetaxel [25]. It provides a proof of concept of targeting HER3 as an effective therapy. However monoclonal antibodies against HER3 have not demonstrated clinical benefits as single agents based on published data as summarized in recent reviews [26,27]. Other approaches targeting HER3 are demanded in clinic.

Perhexiline is an anti-angina drug with the inhibitory properties against the enzyme mitochondrial CPT-1. The plasma concentration of perhexiline in humans within its therapeutic range is about 2 μM [28]. Distribution studies indicate that perhexiline is preferentially distributed in tissues. For example in heart, the perhexiline concentration is at least 10-fold higher than in serum [19,29]. The distribution of perhexiline into tumors versus other tissues is unknown; the concentration of perhexiline in tumors needs to be determined.

The anticancer properties of perhexiline are not well known. It has been reported that perhexiline has an effect on adriamycin-resistant human breast cancer cells. Simultaneous exposure to tamoxifen or perhexiline decreased resistance to adriamycin in a clonogenic assay by an undefined mechanism [30]. Here, we report the anti-breast cancer properties of perhexiline through HER3 degradation and HER3-mediated signaling inhibition. The synergistic inhibitory effect of perhexiline with lapatinib on tumor growth may provide immediate therapeutic benefits for breast cancer patients with drug resistance and metastasis.

In summary, our findings represent a proof of principle by demonstrating the ability of a small molecule to selectively downregulate HER3 but not other epidermal growth factor receptor (EGFR) family members and inhibit downstream signaling in breast cancer cells and tumors. As a clinically used drug, perhexiline may be administrated alone, or in combination with other existing therapies such as lapatinib, to overcome drug resistance and metastasis in cancer treatment. Perhexiline is a first-in-class small molecule that targets HER3. Optimization of its pharmaceutical properties could provide improved derivatives with greater benefit to breast cancer patients. Clinical evaluation of perhexiline and its improved derivatives may directly lead to a new therapy to treat breast cancer and other HER3-dependent cancers.

## Conclusions

Ablating HER3 offers the potential to decrease mortality in breast cancer in patients. Using a novel image-based high-throughput screening assay, we discovered the small molecule drug perhexiline, once used to treat angina, ablates HER3 by promoting HER3 internalization and degradation. This is the first demonstration that HER3 can be targeted with small molecule by eliminating it from the cell membrane. Consistent with this mechanism, perhexiline inhibits HER3-mediated signaling as well as the growth of breast cancer cells *in vitro* and *in vivo*. Our approach overcomes a barrier to develop small molecule therapies targeting HER3. HER3 ablation modulators represent an innovative therapeutic approach to improve survival in breast cancer patients.

## Additional files

Additional file 1: Figure S1. The HER3ΔNLS2-YFP receptor functions similarly as the wild-type HER3-YFP receptor. U2OS cells were transiently transfected with vector, HER3-YFP, or HER3ΔNLS2-YFP expression plasmids. Cells were treated with NRGβ1 for 0 to 30 minutes as indicated, and cell lysates were analyzed for HER3 expression and Akt phosphorylation. Additional file 2: Figure S2. Perhexiline treatment induces downregulation of endogenous HER3 receptors in MDA-MB-468 cells. **(A)** Time course of perhexiline-induced downregulation of endogenous HER3. Cells treated with 10 μM perhexiline for the indicated time were analyzed for endogenous HER3 and EGFR expression. β-actin was used as a loading control. **(B)** Quantification of HER3 and EGFR protein expression following perhexiline treatment. Western blots shown in **(A)** were quantified by normalizing to β-actin. **(C)** Dose-dependent effect of perhexiline on HER3 expression. Cell lysates prepared from cells treated with different concentrations of perhexiline for 8 hours were analyzed for the expression of HER3 and EGFR. **(D)** Quantification of HER3 and EGFR protein expression following perhexiline treatment. Western blots shown in **(C)** were quantified by normalizing to β-actin. **(E)** Dose-dependent effect of perhexiline on HER3 phosphorylation. Cell lysates prepared from cells treated with different concentrations of perhexiline for 6 hours were analyzed for the phosphorylation of HER3 at Tyr1289 site. Additional file 3: Figure S3. Perhexiline induces trafficking of HER3 to lysosome. U2OS cells expressing HER3-ΔNLS2-YFP were treated with DMSO (i to iii) or perhexiline (iv to vi) and co-localization of HER3ΔNLS2-YFP (green) with lysosome (red) was indicated by arrows. Additional file 4: Figure S4. Perhexiline inhibits breast cancer cell proliferation and functions synergistically with lapatinib. **(A** and **C)** The antiproliferative effects of perhexiline on AU565 **(A)** and BT474 **(C)** breast cancer cells. Cells were treated with different concentrations of perhexiline for 72 hours, and cell viability was measured using the MTS assay. Results represent mean ± SEM of three independent experiments. **(B** and **D)** The synergistic effect of perhexiline and lapatinib on inhibiting proliferation of AU565 **(B)** and BT474 **(D)** cells. Cells were treated with increasing concentrations of perhexiline and lapatinib alone or in combination for 72 hours. MTS assays were performed to measure cell viability. Results are presented as the mean ± SEM. Combinational Index (CI) quantifies the degree of synergism in a drug combination. CI is obtained using the method of Chou and Martin in the software CompuSyn. CI <1 indicates drug synergy. ED50, ED75, ED90 and ED95 are the effective doses at which 50%, 75%, 90% and 95% cells are killed, respectively.

## Abbreviations

BSA: bovine serum albumin
CI: combinational index
CPT-1: mitochondrial carnitine palmitoyltransferase-1
DMSO: dimethyl sulfoxide
ED: effective dose
EGF: epidermal growth factor
EGFR: epidermal growth factor receptor
ERK: extracellular signal-regulated protein kinase
FBS: fetal bovine serum
HER: human epidermal growth factor receptor
MEM: Minimum Essential Medium
NRGβ1: neuregulin β1
PBS: phosphate-buffered saline
PCR: polymerase chain reaction
PI3K: phosphatidylinositol 3-kinase
YFP: yellow fluorescent protein
