# Supplementary figures and images for: Perhexiline promotes HER3 ablation through receptor internalization and inhibits tumor growth
Source: Breast Cancer Res. 2015 Feb 15;17(1):20. doi: 10.1186/s13058-015-0528-9 (PMC4358700; doi:10.1186/s13058-015-0528-9)

## Slide 1
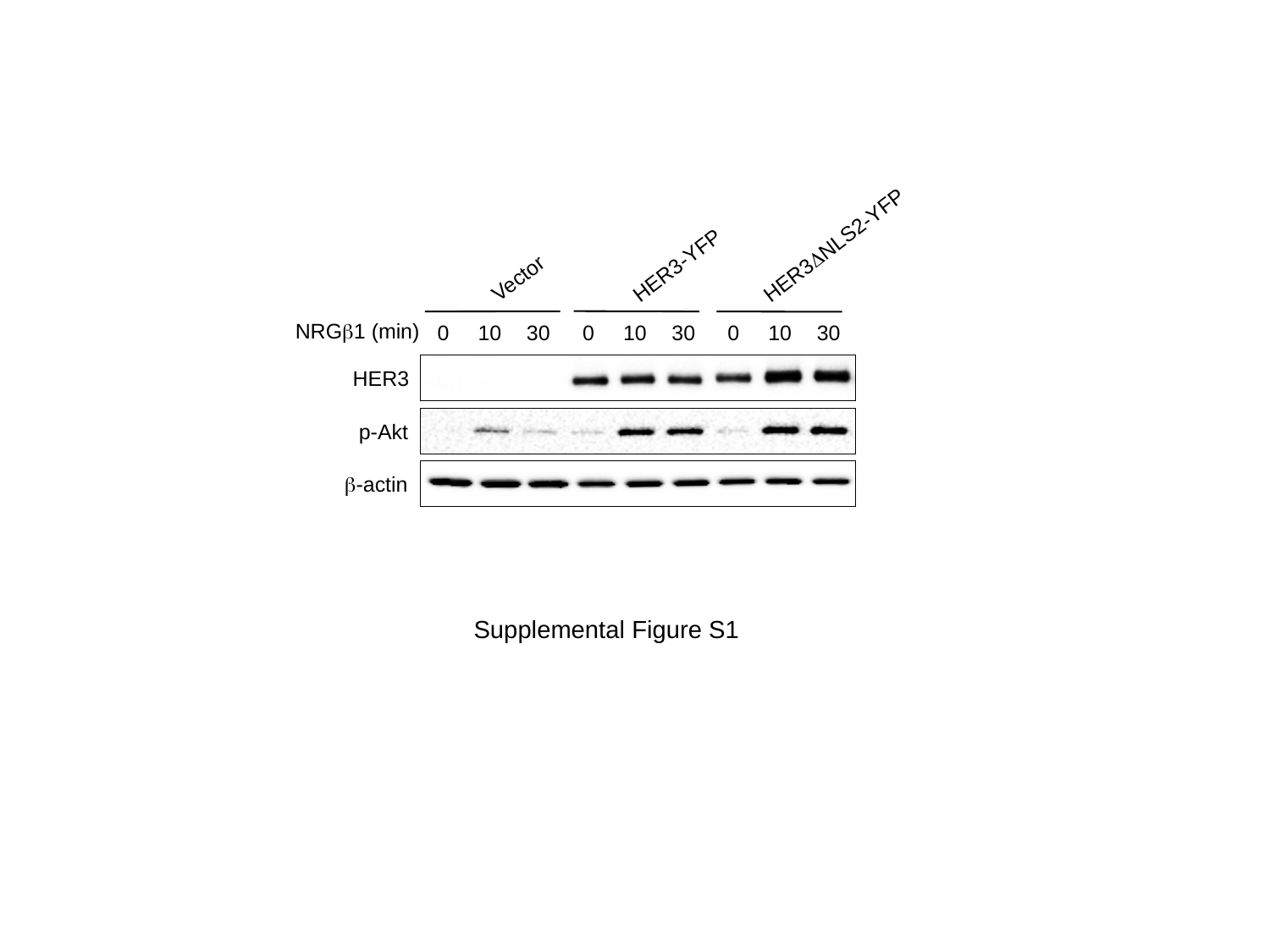

HER3DNLS2-YFP
HER3-YFP
Vector
NRGb1 (min)
0
10
30
0
10
30
0
10
30
HER3
p-Akt
b-actin
Supplemental Figure S1

Supplement: Additional file 1: Figure S1. — The HER3ΔNLS2-YFP receptor functions similarly as the wild-type HER3-YFP receptor. U2OS cells were transiently transfected with vector, HER3-YFP, or HER3ΔNLS2-YFP expression plasmids. Cells were treated with NRGβ1 for 0 to 30 minutes as indicated, and cell lysates were analyzed for HER3 expression and Akt phosphorylation. [file 13058_2015_528_MOESM1_ESM.pptx]

## Slide 1
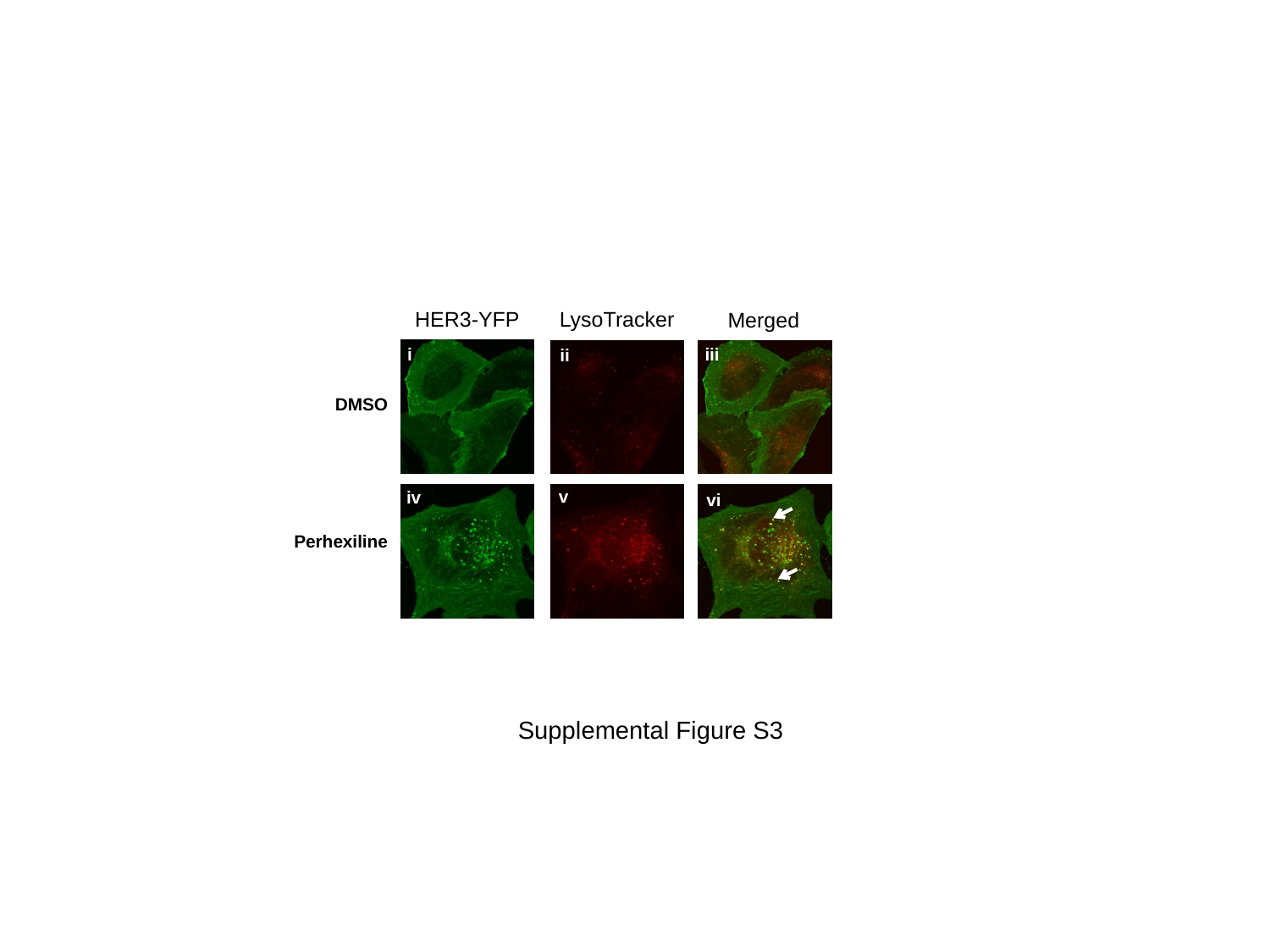

HER3-YFP
LysoTracker
Merged
i
iii
ii
DMSO
v
iv
vi
Perhexiline
Supplemental Figure S3

Supplement: Additional file 3: Figure S3. — Perhexiline induces trafficking of HER3 to lysosome. U2OS cells expressing HER3-ΔNLS2-YFP were treated with DMSO (i to iii) or perhexiline (iv to vi) and co-localization of HER3ΔNLS2-YFP (green) with lysosome (red) was indicated by arrows. [file 13058_2015_528_MOESM3_ESM.pptx]

## Slide 1
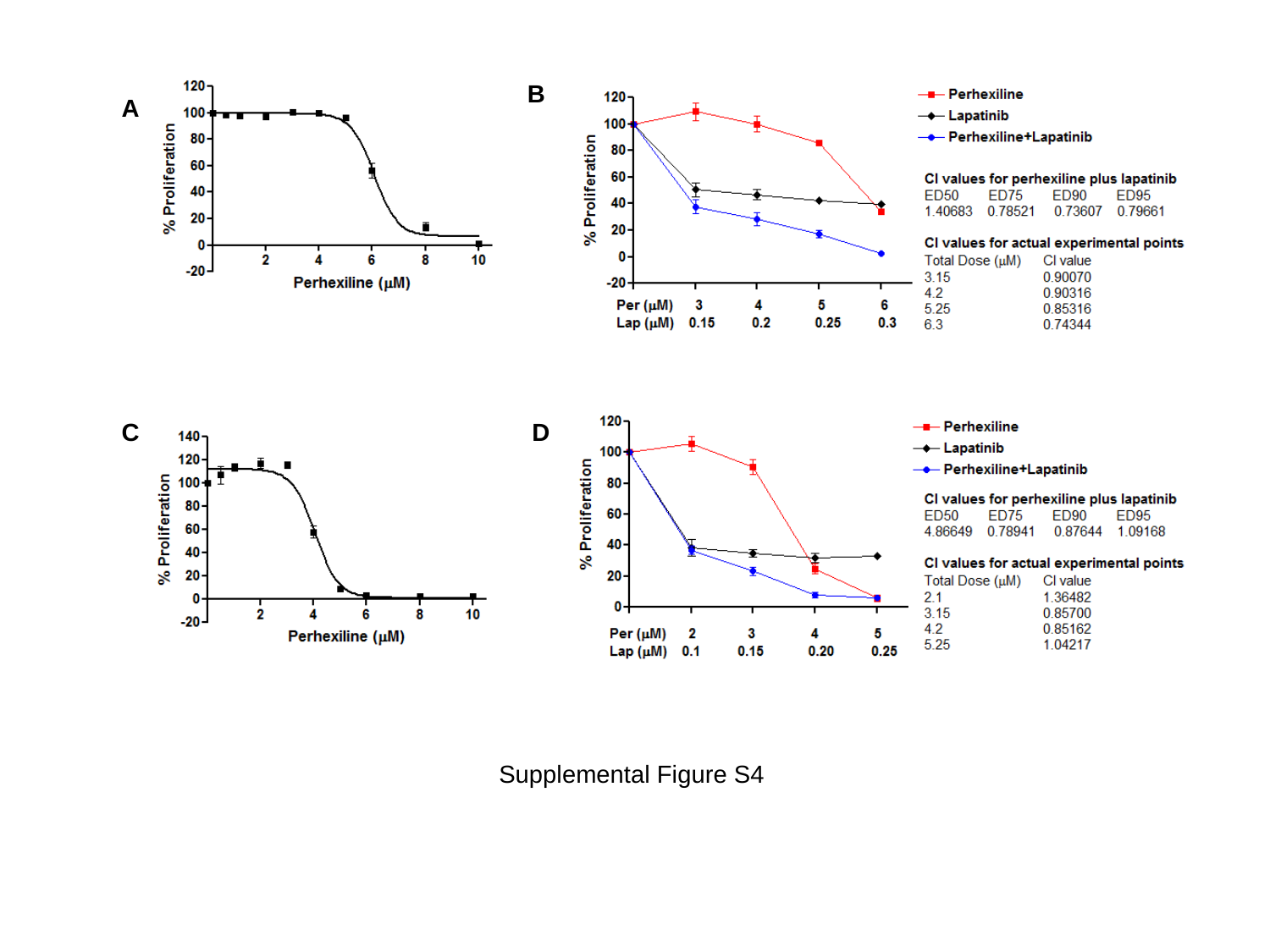

B
A
C
D
Supplemental Figure S4

Supplement: Additional file 4: Figure S4. — Perhexiline inhibits breast cancer cell proliferation and functions synergistically with lapatinib. (A and C) The antiproliferative effects of perhexiline on AU565 (A) and BT474 (C) breast cancer cells. Cells were treated with different concentrations of perhexiline for 72 hours, and cell viability was measured using the MTS assay. Results represent mean ± SEM of three independent experiments. (B and D) The synergistic effect of perhexiline and lapatinib on inhibiting proliferation of AU565 (B) and BT474 (D) cells. Cells were treated with increasing concentrations of perhexiline and lapatinib alone or in combination for 72 hours. MTS assays were performed to measure cell viability. Results are presented as the mean ± SEM. Combinational Index (CI) quantifies the degree of synergism in a drug combination. CI is obtained using the method of Chou and Martin in the software CompuSyn. CI <1 indicates drug synergy. ED50, ED75, ED90 and ED95 are the effective doses at which 50%, 75%, 90% and 95% cells are killed, respectively. [file 13058_2015_528_MOESM4_ESM.pptx]
