# Supplementary material for: Perhexiline promotes HER3 ablation through receptor internalization and inhibits tumor growth
Source: Breast Cancer Res. 2015 Feb 15;17(1):20. doi: 10.1186/s13058-015-0528-9 (PMC4358700; doi:10.1186/s13058-015-0528-9)
Supplement: Additional file 2: Figure S2. — Perhexiline treatment induces downregulation of endogenous HER3 receptors in MDA-MB-468 cells. (A) Time course of perhexiline-induced downregulation of endogenous HER3. Cells treated with 10 μM perhexiline for the indicated time were analyzed for endogenous HER3 and EGFR expression. β-actin was used as a loading control. (B) Quantification of HER3 and EGFR protein expression following perhexiline treatment. Western blots shown in (A) were quantified by normalizing to β-actin. (C) Dose-dependent effect of perhexiline on HER3 expression. Cell lysates prepared from cells treated with different concentrations of perhexiline for 8 hours were analyzed for the expression of HER3 and EGFR. (D) Quantification of HER3 and EGFR protein expression following perhexiline treatment. Western blots shown in (C) were quantified by normalizing to β-actin. (E) Dose-dependent effect of perhexiline on HER3 phosphorylation. Cell lysates prepared from cells treated with different concentrations of perhexiline for 6 hours were analyzed for the phosphorylation of HER3 at Tyr1289 site. [file 13058_2015_528_MOESM2_ESM.pptx]

## Slide 1
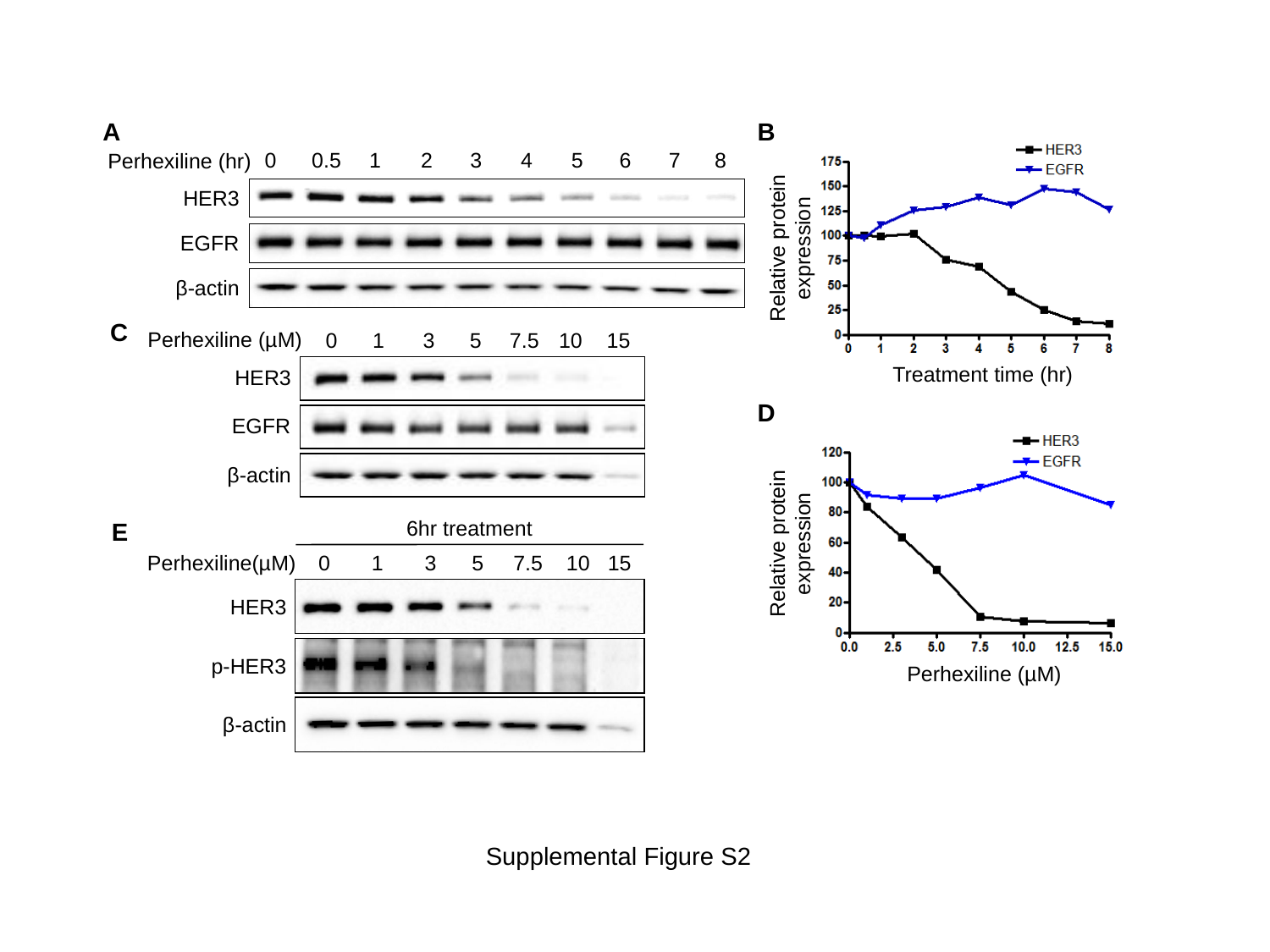

A
B
Relative protein expression
Treatment time (hr)
0
0.5
1
2
3
4
5
6
7
8
Perhexiline (hr)
HER3
EGFR
β-actin
C
Perhexiline (µM)
0
1
3
5
7.5
10
15
HER3
D
EGFR
Relative protein expression
Perhexiline (µM)
β-actin
6hr treatment
E
Perhexiline(µM)
0 1 3 5 7.5 10 15
HER3
p-HER3
β-actin
Supplemental Figure S2
